# Influencing factors of knowledge, attitude and behavior in children’s palliative care among pediatric healthcare workers: a cross-sectional survey in China

**DOI:** 10.1186/s12904-023-01187-4

**Published:** 2023-06-07

**Authors:** Lihui Zhu, Na Zhang, Yaojia Hu, Yi Xu, Tingwei Luo, Yuqiong Xiang, Sishan Jiang, Zhiqiang Zhang, Muhua Chen, Yuee Xiong

**Affiliations:** 1 Nursing Teaching and Research Section, Hunan Cancer Hospital/The Afliated Cancer Hospital of Xiangya School of Medicine, Central South University, Changsha, China; 2 Department of Nursing, Hunan Children’s Hospital, Changsha, China; 3 Department of Ophthalmology, Hunan Children’s Hospital, Changsha, China; 4 Department of Nursing, Hunan University of Chinese Medicine, Changsha, China; 5 Department of Neonatology, Hunan Children’s Hospital, Changsha, China

**Keywords:** Pediatric healthcare workers, Children’s palliative care, Humanistic care

## Abstract

**Background:**

Palliative care has become a key medical field worldwide. Although research relating to adult palliative care is well-established, less is known about children’s palliative care (CPC). Therefore, this study investigated the knowledge, attitude and behavior of pediatric healthcare workers (PHWs) regarding CPC and analyzed the influencing factors for the implementation and development of CPC.

**Methods:**

A cross-sectional survey of 407 PHWs was carried out in a Chinese province from November 2021 to April 2022. The questionnaire consisted of two parts: a general information form and questions on the knowledge, attitude and behavior of PHWs about CPC. Data were analyzed using t-test, ANOVA and multiple regression analysis.

**Results:**

The total score of the PHWs’ knowledge, attitude and behavior about CPC was 69.98, which was at a moderate level. PHWs’ CPC knowledge, attitude, and behavior are positively correlated.The most important influencing factors were working years, highest education, professional title, job position, marital status, religion, grade of hospital (I, II or III), type of medical institution, experience of caring for a terminally ill child/kinsfolk and total hours of CPC education and training received.

**Conclusions:**

In this study, PHWs in a Chinese province had the lowest scores on the knowledge dimension of CPC, with moderate attitude and behavior and various influencing factors. In addition to professional title, highest education and working years, it is also worth noting that the type of medical institution and marital status also affected the score. Continuing education and training of PHWs in CPC should be emphasized by the administrators of relevant colleges and medical institutions. Future research should start with the above-mentioned influencing factors and focus on setting up targeted training courses and evaluating the post-training effects.

**Supplementary Information:**

The online version contains supplementary material available at 10.1186/s12904-023-01187-4.

## Background

Approximately 300,000 new tumors are found in children each year worldwide [1], making them a special category of vulnerable group. Each year in China, 60,000-288,000 new pediatric cancer patients are diagnosed, accounting for 90% of the mortality rate [2]. The clinical manifestations of critical illnesses in dying children are more complex than those of adults, and the lack of clinical treatment experience makes it difficult to diagnose the disease and to predict the prognosis [3]. Not only do these children have a heavy burden of disease, but their families also bear a huge economic, psychological and social burden. When a child is terminally ill and facing death, providing palliative care can reduce suffering and improve the quality of life for the child and family. Children’s Palliative Care (CPC) [4]–[5] refers to a method of care that can effectively improve the quality of life of children with life-threatening illnesses and addressing the problems faced by their families from the time of diagnosis to the end of life, That is, early identification, comprehensive assessment, and planned interventions of the physical, psychosocial and spiritual problems of children and families to alleviate their suffering.

It is estimated that there are more than 21.6 million children and families worldwide in need of specialized palliative care services [6]. There are thousands of hospices in the United States that accept terminally ill children, and more than 50 children’s hospices in the United Kingdom. Countries such as Britain, South Africa and Singapore have established hospice programs through dedicated groups with the support of medical institutions and government [7]–[8]. In China, although palliative care has only been recently introduced, there have been remarkable achievements through decades of development [9]–[10]. Due to the influence of ethnic culture, regional and national policy, as well as the special nature of children’s growth and development roles, CPC is currently only carried out in Changsha, Beijing, Shanghai and Fujian.

As the primary assessor, implementer and educator of palliative care delivery, pediatric healthcare workers’ (PHWs) knowledge, attitude and behavior are the foundations of the work [11]. Only when the knowledge and skills related to CPC are fully developed, can we make behavioral practices for it, so as to provide high-quality CPC services for children and families at the end of life.The theory of knowledge, attitude and practice believes that individuals can eventually develop healthy behaviors by acquiring health knowledge and building positive attitudes. Therefore, attention must be paid to training and raising awareness of knowledge, attitude and behavior.The World Health Organization (WHO) also has requested that palliative care be included in the training of health care workers [12]. As a result, developed countries such as the US and UK have developed multi-level palliative care training courses and related certification procedures [13]. A survey showed that 95% of physicians and over 91% of nursing staff in UK hospices have received specialist training [14]. Thus, training promotes the development of palliative care teams, but at this stage there is a lack of specialized training in CPC. In China, CPC is less developed, and factors such as weak public awareness and insufficient professional staff [15]–[16] hinder its successful implementation. As an important role of PHWs to promote the development of CPC, the only way to improve practice is to understand the meaning of CPC. Therefore, this study aims to understand the level of CPC knowledge, attitude and behavior of PHWs through a cross-sectional survey, analyze the factors affecting these, and provide precise recommendations for targeted CPC training for PHWs.

## Methods

### Design

This study is cross-sectional, and a STROBE statement [17] is used to report this study.

### Procedure

Convenience sampling was used to recruit the sample. First, participants were recruited via a provincial pediatric specialty nurse, a clinical application of family-centered health education system seminar site and 7 hospitals in Zhangjiajie, Yueyang, Hengyang, Chenzhou and Xiangtan. The survey team distributed electronic questionnaires with an online link and/or WeChat QR code linked to a WeChat group. Some of the questionnaires were distributed by linking them to the head of each hospital department, who in turn distributed them to the individual staff in the department. The questionnaire can only be filled out once by the same IP address, and all items could only be submitted after checking. The sample size was calculated using G-Power software v 3.1.9.2 [18] based on a linear multiple regression test with an alpha error of 5%, a power of 95%, and 48 predictors in the model. The required sample size was 260. Accounting for invalid questionnaires, 20% was added to the calculated sample size, meaning the final sample size needed to be larger than 312. All questionnaires were completed using an online survey and using Questionnaire Star (www.wjx.cn), an electronic data collection tool.

### Participants

From November 2021 to April 2022, we conducted a cross-sectional survey of 412 participants. Participants were eligible if they (a) voluntarily participated in the study, (b) had been engaged in pediatric medical or nursing work for more than 1 year; and (c) spoke Chinese as their mother tongue. Participants undergoing training, internship or further education; or who were on maternity, study or sick leave were excluded from the study. This study was approved by the Hunan Children’s Hospital Research Ethics Committee (Reference number: HCHILL-2021-23).

### Measures

An information sheet was designed to gather demographic information from participants including gender, age, highest education, monthly income, working years, professional title, job position, Grade of hospital, type of medical institution, marital status, ethnicity, religion, experience of caring for a terminally ill child/kinsfolk and total hours of CPC education and training received. The survey also included questions on CPC knowledge, attitude and behavior variables.

#### CPC knowledge, attitude and behavior questionnaire

Combining previous studies [19–21], clinical experience of PHWs and group discussion, we prepared a questionnaire about CPC knowledge, attitude and behavior. The questionnaire then underwent Delphi expert consultation with 62 PHKs in a Chinese province to test its reliability and validity. A final total of 48 items were included, including knowledge (15 items), attitude (15 items) and behavior (18 items). The total Cronbach’s α coefficient of the questionnaire was 0.877, and the dimensions of knowledge, attitude and behavior were 0.845, 0.822 and 0.961, respectively. The content validity was 0.893. The knowledge dimension included the definition, recipients of palliative care services, concepts and principles of CPC. The total score of the CPC knowledge section was 100 points, with a score of 5 points for questions 1–10 and 10 points for questions 11–15. Higher scores indicated better knowledge of CPC. The attitude dimension included attitude towards the implementation of CPC, attitude towards attending death education or hospice-related courses and attitude towards communicating and cooperating with children and their families. A Likert 5-point scale was used for each item, and the score was 1–5 in the order: “strongly disagree, basically disagree, uncertain, somewhat agree and strongly agree”. Higher scores indicated a more positive attitude toward CPC among the study participants. The behavioral dimensions included pain intervention, comfort intervention and meeting the reasonable needs of children and their families. A Likert 5-point scale was used to score 1–5 points in the order: “rarely, occasionally, sometimes, often and always”, with higher scores indicating better CPC behavior. Knowledge, attitude, behavior and the total score of the three were calculated using standard score, which was average score/total score × 100%.The total score of the questionnaire and of each dimension was interpreted as: < 60 poor, 60 to 85 moderate, > 85 good [22].

### Data analysis

SPSS v 26.0 was used to conduct data analysis and the significance level was *P* < 0.05. Descriptive statistics were calculated to describe participant characteristics and to summarize the data.

To determine the factors affecting PHW knowledge, attitude and behavior, univariate analyses were conducted. Significant factors were then subjected to multiple regression analysis. T-test and ANOVA were used to compare the scores for each factor according to demographic and other characteristics of PHWs. Multiple regression analysis was applied to assess the influence of major variables on the level of PHWs CPC knowledge, attitude and behavior. In multiple regression analysis, the knowledge, attitude and behavior scores for each factor were used as dependent variables, and variables that were found to be statistically significant by univariate analysis were used as independent variables.

## Results

### Characteristics of the study population

A total of 412 questionnaires were sent out and 407 were returned, an effective recovery rate of 98.79%. Among the 407 healthcare workers, 292 (71.7%) were female, 110 (27.0%) had a junior college degree, 202 (49.6%) had a bachelor’s degree, 87 (21.4%) had a master’s degree, and 8 (2.0%) had a doctorate degree. There were 131 doctors (32.2%) and 276 nurses (67.8%). Other general data characteristics are shown in Table 1.

**Table 1:** Results of univariate analysis of general information and PHW knowledge and belief scores(${\bar x}$ ± s, n = 407)

Variable	Number of people	Constituent ratio (%)	Knowledge	Attitude	Behavior
Age (year)					
≤ 29	94	23.1	57.77 ± 14.01	63.21 ± 7.22	64.20 ± 20.77
30–39	200	49.1	57.20 ± 14.24	62.87 ± 6.34	62.19 ± 20.34
40–49	90	22.1	60.33 ± 14.22	63.13 ± 5.88	65.68 ± 22.85
≥ 50	23	5.7	63.91 ± 13.23	64.48 ± 5.55	71.35 ± 23.28
F			2.270	0.449	1.599
*P*			0.080	0.718	0.189
Highest education					
Junior college	110	27.0	55.09 ± 15.93	61.53 ± 7.27	59.07 ± 21.71
Undergraduate	202	49.6	59.46 ± 13.36	63.31 ± 5.94	65.24 ± 20.76
Postgraduate	87	21.4	59.77 ± 13.31	64.46 ± 5.84	66.92 ± 21.26
Doctoral candidate	8	2.0	65.63 ± 9.03	66.00 ± 6.02	70.38 ± 15.36
F			3.494	4.360	3.148
*P*			0.016*	0.005**	0.025*
Monthly income (yuan)					
<5000	51	12.5	53.04 ± 14.53	61.67 ± 8.34	60.27 ± 22.69
5000–8000	176	43.0	57.81 ± 14.04	62.48 ± 6.05	59.50 ± 21.26
8001–10,000	128	31.7	59.65 ± 14.24	64.13 ± 6.39	67.41 ± 19.35
< 10,000	52	12.8	62.59 ± 12.89	64.06 ± 4.93	74.02 ± 19.59
F			4.476	2.943	8.570
*P*			0.004*	0.033*	< 0.001**
Working years					
≤ 5	89	21.9	55.96 ± 13.89	62.57 ± 7.89	58.40 ± 19.86
6–10	111	27.3	59.09 ± 13.67	63.09 ± 6.14	59.18 ± 22.44
11–20	156	38.3	57.95 ± 15.04	63.43 ± 6.09	68.43 ± 20.32
≥ 21	51	12.5	62.55 ± 12.50	63.02 ± 4.98	70.25 ± 19.19
F			2.499	0.339	8.112
*P*			0.059	0.797	< 0.001**
Professional title					
Junior	126	31.0	54.52 ± 13.92	61.89 ± 7.61	60.56 ± 19.91
Intermediate	194	47.7	59.28 ± 14.32	63.32 ± 5.95	62.61 ± 20.61
Deputy senior	77	18.9	61.95 ± 12.57	64.27 ± 4.98	70.29 ± 22.85
Senior	10	2.5	63.00 ± 17.83	64.90 ± 6.45	83.60 ± 18.19
F			5.503	2.714	6.759
*P*			0.001**	0.045*	< 0.001**
Job position					
Doctor	131	32.2	56.26 ± 14.26	62.17 ± 7.22	60.38 ± 23.06
Nurse	276	67.8	59.42 ± 14.09	63.54 ± 5.94	65.63 ± 20.13
t			− 2.106	− 2.026	− 2.345
*P*			0.036*	0.043*	0.020*
Grade of hospital					
Grade 1 hospital	6	1.5	45.00 ± 20.00	55.67 ± 5.05	53.33 ± 17.51
Grade 2 A hospital	100	24.6	56.30 ± 12.74	63.60 ± 6.08	61.46 ± 25.45
Grade 2B hospital	20	4.9	51.25 ± 14.86	59.30 ± 8.59	56.65 ± 22.34
Grade 3 A hospital	216	53.1	60.35 ± 13.65	63.57 ± 6.53	66.81 ± 18.77
Grade 3B hospital	65	16.0	58.62 ± 15.92	62.62 ± 5.01	61.46 ± 20.75
F			4.303	4.461	2.552
*P*			0.002**	0.002**	0.039*
Type of medical institution					
General hospital	140	34.4	56.29 ± 16.19	61.81 ± 6.96	60.84 ± 22.23
Specialized hospital	267	65.6	59.51 ± 12.94	63.78 ± 5.99	65.57 ± 20.54
t			− 2.188	-2.972	− 2.148
*P*			0.029*	0.003**	0.032*
Marital status					
Unmarried	102	25.1	55.44 ± 14.47	61.12 ± 7.56	58.84 ± 20.29
Married	284	69.8	59.49 ± 13.62	63.79 ± 5.78	65.78 ± 21.62
Divorced	14	3.4	61.43 ± 14.73	63.50 ± 6.64	64.36 ± 18.14
Widowhood	7	1.7	51.42 ± 24.95	63.14 ± 7.22	62.86 ± 14.31
F			2.845	4.480	2.714
*P*			0.037*	0.004**	0.045*
Religion					
Nothing	374	91.9	58.68 ± 14.02	62.49 ± 60.3	63.16 ± 20.45
Buddhism	33	8.1	55.30 ± 16.05	70.03 ± 6.53	72.85 ± 27.50
t			− 1.309	6.840	2.530
*P*			0.191	< 0.001**	0.012*
Experience of caring for a terminally ill child/kinsfolk					
Have	159	39.1	61.67 ± 12.67	64.62 ± 6.15	68.77 ± 18.59
Do not have	248	60.9	56.31 ± 14.75	62.13 ± 6.39	60.84 ± 22.25
t			3.772	3.894	3.733
*P*			< 0.001**	< 0.001**	< 0.001**
Total hours of CPC education and training received					
0 credit hours	267	65.6	55.87 ± 13.62	61.98 ± 6.41	59.78 ± 19.62
1–5 credit hours	95	23.3	61.40 ± 14.11	64.45 ± 5.85	68.78 ± 21.73
6–10 credit hours	29	7.1	65.16 ± 15.36	66.48 ± 6.53	78.97 ± 21.08
11–15 credit hours	10	2.5	67.50 ± 8.58	67.50 ± 4.79	71.80 ± 25.97
16 and above credit hours	6	1.5	68.33 ± 14.02	64.33 ± 4.13	73.00 ± 20.89
F			7.104	6.881	8.943
*P*			< 0.001**	< 0.001**	< 0.001**

Note: *P < 0.05, **P < 0.01

### Score of CPC knowledge, attitude and behavior for PHWs

The mean total scores across 407 PHWs for knowledge, attitude and behavior were 58.4, 63.1 and 63.9 ; and the mean standard scores were 58.4, 84.1 and 71.4, respectively. The score for attitude and behavior was medium, and the score for knowledge was poor. The scores for each dimension and item are shown in Tables 2 and 3.

**Table 2:** Score of CPC knowledge, attitude and behavior of PHKs (n = 407)

Dimension	Score (x ± s)	Standard score (x ± s)
Total knowledge score	58.40 ± 14.20	58.40
Total attitude score	63.10 ± 6.01	84.13
Total behavior score	63.94 ± 21.23	71.04
Total score of knowledge, attitude and behavior	185.44 ± 28.32	69.98

**Table 3:** PHWs scores for each entry of knowledge, attitude and behavior in CPC (n = 407)

Item	Minimum value	Maximum value	Average score
Knowledge			
Definition	0	5	1.22 ± 0.32
Purpose	0	5	1.94 ± 0.92
Service Users	0	5	4.15 ± 1.88
Idea	0	5	4.45 ± 1.57
Principle	0	5	4.00 ± 1.99
Connotation	0	5	4.42 ± 1.59
WHO three-step pain relief principles	0	5	2.49 ± 2.33
Third-order painkillers	0	5	1.42 ± 0.44
Pain management	0	5	2.96 ± 2.46
Child comfort care	0	5	2.35 ± 1.64
Possible symptoms	0	10	8.57 ± 3.49
Practice content	0	10	8.47 ± 3.59
Features	0	10	6.68 ± 4.71
Objective	0	10	5.99 ± 4.90
Revelation	0	10	4.64 ± 3.14
Attitude			
Attitudes toward Palliative care	1	5	4.67 ± 0.64
Attitudes toward the child’s death and near death	1	5	4.52 ± 0.69
Attitudes toward palliative care in their hospital	1	5	3.81 ± 1.01
Perceptions of talking about death	1	5	3.11 ± 1.21
Perceptions of transferring the emotions of the child	1	5	3.91 ± 1.04
Perceptions of active family participation in the care of the child’s life	1	5	4.68 ± 0.57
Attitude of care for the child’s family	2	5	4.68 ± 0.56
Perceptions of family care and assistance for terminally ill children	2	5	4.76 ± 0.49
Perceptions of family members maintaining normal attitudes and behaviors in the presence of the child	1	5	4.28 ± 0.89
Perceptions of the leading end-of-life decision maker being the child and family	1	5	4.17 ± 0.89
Perceptions of terminally ill children’s verbal expression of their feelings	2	5	4.39 ± 0.72
Perceptions of truthfulness in informing terminally ill children of their condition	1	5	3.48 ± 1.09
Perceptions of attending death education or palliative care study	1	5	4.51 ± 0.66
Views on home CPC whose costs are covered by health insurance	1	5	4.36 ± 0.79
Attitudes toward the importance the government places on CPC	1	5	3.76 ± 1.02
Behavior			
Pain Management	1	5	2.86 ± 1.34
Oral Care	1	5	3.02 ± 1.33
Skin Care	1	5	3.35 ± 1.33
Nutritional Care	1	5	3.18 ± 1.27
Respiratory Care	1	5	3.55 ± 1.27
Sleep Care	1	5	3.29 ± 1.27
Psychological care	1	5	3.41 ± 1.23
Think Different	1	5	3.48 ± 1.16
Proactive assessment of attitudes toward death	1	5	3.18 ± 1.26
Will help to gain knowledge about death	1	5	3.03 ± 1.29
Will assist in fulfilling realistic and feasible wishes	1	5	3.19 ± 1.29
Can encourage family companionship and facilitate communication between the two parties	1	5	3.38 ± 1.26
Respect for ethnic and cultural specific needs	1	5	3.20 ± 1.29
Will ask for input when developing care plans	1	5	3.41 ± 1.27
Able to assess the psychological state of the family and provide support	1	5	3.29 ± 1.26
Can encourage and assist families in finding appropriate ways to say goodbye to their children	1	5	3.22 ± 1.34
Can encourage families to send their condolences in an appropriate way	1	5	3.02 ± 1.37
Follow-up support for resident mourners after the death of the child	1	5	2.26 ± 1.25

### Results of correlation analysis of PHWs’ CPC knowledge, attitude and behavior

Pearson correlation analysis showed a positive correlation (*p* < 0.05) between PHWs’ CPC knowledge, attitude, and behavior scores, with r values of 0.124, 0.154, and 0.233, as shown in Table 4.

**Table 4:** Correlation analysis of knowledge, attitude and behavior of PHWs in CPC

Dimension	Knowledge	Attitude	Behavior
Knowledge	1.000	0.124*	0.154**
Attitude	0.124*	1.000	0.233**
Behavior	0.154**	0.233**	1.000

Note: *P < 0.05, **P < 0.01

### Univariate analysis of PHWs CPC knowledge, attitude and behavior scores

Table 1 presented CPC knowledge, attitude and behavior for different general characteristics. CPC knowledge, attitude and behavior in participants with a doctoral degree were significantly higher than in those below this level (p = 0.016, p = 0.005, p = 0.025). CPC knowledge, attitude and behavior in participants with a monthly income > 5000 yuan was significantly higher than in those who earned < 5000 yuan (p = 0.004, p = 0.033, p < 0.001). CPC knowledge, attitude and behavior in those working in a hospital of Grade 2 A or above was significantly higher than in those working at a hospital below Grade 2 A (p = 0.002, p = 0.002, p = 0.039). CPC knowledge, attitude and behavior showed a significant difference with professional title (p = 0.001, p = 0.045, p < 0.001), job position (p = 0.036, p = 0.043, p = 0.020), type of medical institution (p = 0.029, p = 0.003, p = 0.032), marital status (p = 0.037, p = 0.004, p = 0.045), experience caring for a terminally ill child/kinsfolk (p < 0.001, p < 0.001, p < 0.001) and different total hours of CPC education and training (p < 0.001, p < 0.001, p < 0.001). In addition, there was a significant difference in attitude and behavior in different religion of PHWs (p < 0.001, p = 0.012). Finally, CPC behavior in participants with ≥ 21 working years was significantly higher than in those with < 21 working years (p < 0.001). However, other characteristics of PHWs had no obvious impact (p > 0.05).

### Multiple regression analysis of PHWs CPC knowledge attitude and behavior scores

The independent variables that were statistically significant in the univariate analysis were analyzed by multiple linear regression (α in = 0.05, α out = 0.10), and were assigned as shown in Table 5. Religion was the influencing factor of CPC attitude, working years was the influencing factors of CPC behavior, and highest education, job position, experience caring for a terminally ill child/kinsfolk and total hours of CPC education and training received were the influencing factors of CPC knowledge, attitude and behavior. The regression coefficients were statistically significant (*P* < 0.05), as shown in Table 6.

**Table 5:** Independent variable assignment table

Independent variable	Variable assignment
Highest education	1 = Junior college below; 2 = Undergraduate; 3 = Postgraduate; 4 = Doctoral candidate
Professional title	1 = Junior; 2 = Intermediate; 3 = Deputy senior; 4 = Senior
Job position	1 = Doctor; 2 = Nurse;
Grade of hospital	1 = Grade IIIA; 2 = Grade 3B; 3 = Grade 2 Class A; 4 = Grade 2B; 5 = Grade 1
Type of medical institution	1 = General hospital; 2 = Specialized hospital
Marital status	1 = Unmarried; 2 = Married; 3 = Divorced; 4 = Widowed
Working years	1 = ≤ 5; 2 = 6–10; 3 = 11–20; 4 = ≥ 21
Religion	0 = Nothing; 1 = Buddhism
Experience of caring for a terminally ill child/kinsfolk	0 = Have; 1 = Not have
Total hours of CPC education and training received	0 = 0 credit hours; 1 = 1–5 credit hours; 2 = 6–10 credit hours; 3 = 11–15 credit hours; 4 = 16 and above credit hours

**Table 6:** Multiple linear regression analysis of PHWs knowledge, attitude and behavior about CPC (n = 407)

Dependent variable	Independent variable	b	Sb	b’	t	*P*
Knowledge [1]	Constant	49.207	5.740	-	8.572	< 0.001
	Highest education	2.100	1.027	0.112	2.044	0.042
	Professional title	2.311	0.969	0.125	2.385	0.018
	Job position (Doctor = reference group)					
	Nurse	3.374	1.581	0.111	2.134	0.033
	Grade of hospital	− 1.504	0.690	− 0.110	-2.179	0.030
	Experience of caring for a terminally ill child/kinsfolk	− 3.520	1.374	− 0.121	-2.562	0.011
	Total hours of CPC education and training received	3.423	0.788	0.204	4.342	< 0.001
Attitude [2]	Constant	67.660	3.221	-	21.003	< 0.001
	Highest education	1.335	0.444	0.158	3.008	0.003
	Job position (Doctor = reference group)					
	Nurse	2.332	0.686	0.170	3.399	0.001
	Type of medical institution (General hospital = reference group)					
	Specialized hospital	2.021	0.627	0.150	3.223	0.001
	Marital status (Unmarried = reference group)					
	Married	1.211	0.518	0.107	2.337	0.020
	Religion	− 6.855	1.078	-0.292	-6.359	< 0.001
	Experience of caring for a terminally ill child/kinsfolk	− 1.329	0.599	-0.101	-2.219	0.027
	Total hours of CPC education and training received	0.750	0.350	0.100	2.139	0.033
Behavior [3]	Constant	51.853	11.082	-	4.679	< 0.001
	Highest education	3.416	1.551	0.122	2.203	0.028
	Job position (Doctor = reference group)					
	Nurse	5.575	2.373	0.123	2.349	0.019
	Working years	3.133	1.488	0.143	2.106	0.036
	Experience of caring for a terminally ill child/kinsfolk	− 4.222	2.060	-0.097	-2.050	0.041
	Total hours of CPC education and training received	4.271	1.206	0.172	3.542	< 0.001

## Discussion

### PHWs CPC knowledge, attitude and behavior scores were moderate overall, with the highest scores on the attitude dimension, to be further improved through training

This study showed that the total standardized score of 69.98 for knowledge, attitude and behavior about CPC was at a moderate level.The knowledge dimension scored the lowest, followed by behavior and then attitude. It is possibly related to the relatively recent introduction of CPC in China and the serious lack of continuing education and training. In 2010, the first hospice for children in China, Butterfly House, was established in Changsha First Welfare Institution in Hunan Province, followed by special ward schools and children’s palliative outpatient clinics. Although hospice-related legal policies have recently become more widespread in China, there is a lack of localized policy and institutional guidance. CPC is only carried out in individual children’s hospitals in provincial capital cities and has not been popularized throughout the province. In addition, the service model is single, consisting mainly of medical and nursing staff from relevant departments, most of whom have not received professional training and therefore lack knowledge [23].

The knowledge dimension scores are consistent with previous findings [23–25], but there is room for PHWs to improve on issues such as the definition and purpose of CPC as well as the management of pain and other symptoms. Jessop S et al [26] explored the barriers to palliative care encountered by PHWs caring for seriously ill children and found that inadequate pain management and hospice education were common; this paper was widely recognized and played an important role in the field after publication. Therefore, we need to further strengthen the training of PHWs in CPC, especially to popularize the concept and improve management of pain and related symptoms.The present study found better scores on the attitude dimension, which was higher than previous results [27]. Caring for terminally-ill children is an emotive subject, and the whole family will be in grief. As important coordinators, PHWs will need to show empathy through close communication with the child or their families. In addition, nurses often feel guilty when withdrawing life-sustaining treatment measures from terminally ill children [28]. Doctors still have to treat terminally ill children according to the wishes of their families when there is no hope of saving them, which increases the suffering of the children and puts PHWs in conflict. Therefore, PHWs aim to provide better palliative care for the children so that they can finish their final journey peacefully [29], and therefore the attitude of CPC is positive.

However, the level of CPC behavior is moderate, which is related to the preliminary level of CPC in China, the lack of professional practice guidelines and standards, insufficient medical and nursing staffing and a lack of unified team management and standardized operational procedures [30]–[31]. The lowest behavioral score was for “Do you provide follow-up support to the bereaved person after the death of the child”, which is not consistent with the results of Western studies [32]. In recent years, countries have been paying more attention to the management of bereavement follow-up, expanding the target group from parents to siblings and gradually increasing the duration of follow-up visits.The National Comprehensive Cancer Network (NCCN) Practice Guidelines [11] also suggest care of children’s families after death as a necessary part of oncology care. However, only a few hospitals in mainland China provide follow-up management for the bereaved, and the follow-up management system is incomplete. Therefore, after the death of a terminally ill child, health care professionals must improve follow-up of the family and provide bereavement counseling. The medical management department must therefore carefully construct the CPC team, build a multidisciplinary collaborative CPC team, improve and standardize practice guidelines and work systems and strengthen relevant knowledge and skills training.

### PHWs’ CPC knowledge, attitude, and behavior are positively correlated

The results of this study showed a positive correlation between PHWs’ CPC knowledge, attitude, and behavior, indicating that the better PHWs knowledge of CPC, the more positive their attitude towards CPC and the better their behavioral compliance. In line with the philosophy of the Knowledge-attitude-practice model [33]. This is consistent with the research results of ACHORA S and KIM S H [34–35]. Their study showed that enhanced knowledge education of nurse can increase willingness to provide palliative care and promote related behaviors.Therefore, enhanced training and learning of PHWs in CPC and increased beliefs in CPC can facilitate their implementation of CPC behaviors. However, there are many confounding factors that affect the knowledge, attitude, and behavior of PHWs, so attitudes are not always positive and behaviors are not always implemented when the level of knowledge is high. For example, the severity of the child’s illness and the workload of the PHWs can affect their attitudes or behaviors in conducting CPC. Therefore, hospitals can take targeted measures in combination with their own relevant factors.

### The level of PHWs knowledge attitude and behavior about CPC is influenced by multiple factors

This study found that PHWs knowledge scores of CPC were related to their professional title and grade of hospital (I, II or III), attitude scores were related to marital status, type of medical institution and religion, behavior scores of PHWs were positively related to working years.The results of this study also showed that the level of PHWs knowledge, attitude and behavior were related to the highest education level, professional title, experience caring for a terminally ill child/kinsfolk, and total hours of CPC education and training.The higher the professional title the better their knowledge, in general agreement with previous findings [36]. Accumulating qualifications and experience increases the probability of encountering relevant events in the workplace as well as opportunities for learning and training. This results in more systematic and advanced CPC received, improving the knowledge base of CPC. The higher the grade of the hospital, the better the knowledge of CPC, which is consistent with the results of many similar studies [36–38]. The higher the grade of the hospital, the more obvious the geographical advantage. For example, provincial hospitals’ regulations, cultural environment and medical resources are better than those of municipal hospitals, and they accept more critically ill children; therefore PHWs can have more opportunities to carry out palliative care. The more resources available, the more clinical experience, the more exposure to critically ill children and the earlier the introduction and implementation of CPC; and therefore the better the knowledge base of CPC.

The most important finding in the present study was that attitude scores were related to marital status, type of medical institution and religion. This is consistent with a previous study [39] but for different reasons. The population surveyed in this study was PHWs in Hunan Province, but the subjects of previous study were Healthcare Workers from hospitals in Changsha City. Therefore, the questionnaire items in this study focused on content related to CPC, while previous studies have focused on comprehensive palliative care. Married PHWs can empathize more with families during treatment and care. PHWs in specialized hospitals had higher attitude scores toward CPC than general hospitals, probably because many of the pediatric patients they treat are critically ill. When children cannot be treated for various reasons, it has become an important task to continuously explore ways to improve their quality of life. With increasing awareness of CPC, the attitude is also more positive. However, there is only one pediatric department in the general hospital. The number of children admitted is limited, and the type of disease is relatively simple. Therefore, there are few opportunities to practice CPC and the understanding is insufficient. The higher attitude score of PHWs who practiced a religion is consistent with the mainland study [40], but contradicts the findings of Korean scholars [41]. The reason for this is that different countries have different national conditions, religious cultures and ideas. Scholars in the Chinese Mainland believe that the spirit of philanthropy and compassion advocated and practiced by religious culture caters to the concept of palliative care and has a certain role in promoting palliative care. Therefore, religiously committed PHWs might be better able to provide understanding, compassion and care to terminally ill children and their families at the spiritual and psychological levels under the dictates of their faith.The CPC behavior scores of PHWs were positively related to working years, due to greater clinical experience in treating and nursing dying children. Therefore, the greater the number of working years, the better the ability to practice CPC behavior.

The results of this study also showed that the level of PHWs knowledge, attitude and behavior were related to the highest education level, professional title, experience caring for a terminally ill child/kinsfolk, and total hours of CPC education and training. The higher the education level, the better the knowledge, attitude and behavior of PHWs, which is consistent with the results of a previous study [42]. Highly educated PHWs can take the initiative to learn according to their own needs, expand their knowledge field, access the most advanced knowledge and better understand the meaning and significance of CPC. As a result, they have a more positive attitude and motivation to participate in CPC, and therefore have higher levels of knowledge, attitude and behavior. The higher scores of pediatric nurses than physicians are not consistent with the results of related studies at home and abroad [25, 43]. This is possibly because the sample size of nurses surveyed was larger and the purpose of CPC is care. As the main caregivers, nurses have the closest contact with dying children and their families, which can improve empathy. Therefore, nurses could be more willing to learn CPC so they can be more proactive in their work. PHWs with previous care experience had higher scores of knowledge, attitude and behavior, which is consistent with the results of many studies [41, 44−45]. During the caregiving process, they actively pay attention to relevant knowledge and accumulate theoretical and practical experience. As a result, they will be more skilled in treating and caring for terminally ill children, will have greater self-confidence, and will have a more positive attitude and behavior in palliative care. This study also showed that those with longer hours of education and training had higher scores of knowledge, attitude and behavior, which is consistent with the findings of Slater [46], Muckaden [47] and Weaver [48]. Systematic and standardized education and training can teach theoretical and practical content in a holistic manner, which makes the role of healthcare workers more clearly perceived and improves their self-efficacy, and they will actively practice in clinical work. It can be seen that education and training is one of the main ways to improve the knowledge, attitude and behavior of PHWs.

## Strengths and limitations

There were several strengths to our study. First, it is one of the first to report CPC knowledge, attitude and behavior among PHWs. Secondly, the area we investigated was the first province in mainland China where CPC was introduced, and the results have some reference significance in China. Additionally, we not only investigated the current situation of the level of knowledge, attitude and behavior, but also performed a factor analysis. Limitations also need to be acknowledged. The sample source in this study was limited and participants were all from one province. Therefore, differences in knowledge, attitude and behavior among PHWs in different regions and provinces need to be explored. In addition, this study used a scaled questionnaire, and the accuracy and scientific validity of this questionnaire need to be further tested. Finally, the Chinese cultural background of the participants may limit the generalizability of the results to other target populations from different cultures. It is suggested that a larger sample and multi-center research study of the provinces could follow. This could provide a reference for training PHWs on CPC in China.

## Conclusion

The total score of the PHWs’knowledge, attitude and behavior about CPC was at a moderate level. And knowledge, attitude and behavior are positively correlated. working years, highest education, professional title, job position, marital status, religion, grade of hospital (I, II or III), type of medical institution, experience of caring for a terminally ill child/kinsfolk and total hours of CPC education and training received were the mainly influencing factors in this study.Therefore, it is recommended that institutions and healthcare administrators take these influencing factors into account when setting up CPC training courses. It is hoped that professional, standardized, systematic and procedural CPC training courses will be set in the future, and the effectiveness of the training evaluated afterwards.

## Electronic supplementary material

Below is the link to the electronic supplementary material.

Supplementary Material 1

## Data Availability

The datasets generated and analyzed during the current study are not publicly available owing to institutional policy but are available from the corresponding author on reasonable request.
