# Supplementary material for: Influencing factors of knowledge, attitude and behavior in children’s palliative care among pediatric healthcare workers: a cross-sectional survey in China
Source: BMC Palliat Care. 2023 Jun 7;22:67. doi: 10.1186/s12904-023-01187-4 (PMC10245604; doi:10.1186/s12904-023-01187-4)
Supplement: Supplementary file 1 — Supplementary Material 1 [file 12904_2023_1187_MOESM1_ESM.docx]

**A survey of the current status of palliative care among pediatric health care providers**

Dear Colleagues:

Hello!

Thank you for taking part in this survey during your busy schedule. The purpose of this survey is to understand the current state of hospice perceptions, beliefs, and behaviors among pediatric health care professionals. Your responses are for scientific research purposes only, and all information you provide will be kept strictly confidential. Please make sure you fill out each question carefully and do not miss it. If you feel that none of the answers to a question are appropriate, choose the answer that is closest to your perceptions; there is no right or wrong answer to a question. Please fill out the questionnaire according to your real thoughts and clinical practice. Thank you for your support and cooperation!

This questionnaire is divided into two parts: the first part is a general information questionnaire. The second part is about hospice perceptions, beliefs/attitudes and behaviors.

Hunan Children Hospital

Would you like to participate in the survey? □Yes □No

**Part 1 General Information Questionnaire**

1. Your gender: □Male □Female
2. Your age:
3. Your Highest Education: □Specialized degree and below □Bachelor's degree □Master degree □Doctoral degree
4. Your monthly financial income: <5000 RMB □5000-8000 RMB □8001-10,000 RMB □>10000 RMB
5. Your working years: □≦5 years □6-10 years □11-20years □≧21 years
6. Your title:□Primary □Intermediate □Deputy High □Positively High
7. Job position: □Doctor □Nurse □Medical Technicians □Management
8. Your hospital level: □Grade IIIA Hospital □Grade IIIB Hospital □Grade IIA Hospital □Grade 2B Hospital □Grade I Hospital
9. Category of your medical institution: □General Hospital □Specialized Hospitals
10. Your marital status: □Unmarried □Married □Divorce □bereaved spouse
11. Your Nation:
12. Do you have a religious affiliation: □Yes (Please specify) □No
13. Experience caring for a terminally ill child/loved one: □Yes □No
14. Have received education and training in hospice care: □Yes(If you choose yes, then do 15 questions) □No
15. Total hours of hospice education and training received (1 credit hour = 45 minutes): □1-5 credit hours □6-10 credit hours □11-15 credit hours □16 or more credit hours

**Awareness of palliative care**

[A total of 15 questions, of which 1-10 questions single choice, 11-15 questions multiple choice](K1)

1. The definition of hospice care is incorrect (D)

A. Hospice service targets include children with life-limiting illnesses

B. Hospice is a multidisciplinary, collaborative model of practice centered on the terminally ill child and family

C. Hospice care is provided in the hospital, at the child's home, or in a community service center

D. Hospice services are provided to terminally ill children only

E. The main components of hospice care include pain and other symptom control, comfort care, psychological, spiritual and social support, etc.

2. Hospice care is based on (D)(K2)

A. Treatment-based B. Prolongation of survival time based C. Cure-based D. Symptomatic treatment-based E. Life care-based

3. The target of hospice care is (C)(K3)

A. terminally ill children

B. medical and nursing staff

C. terminally ill children and their families

D.Acutely ill children

E. Chronically ill children

4. The philosophy of hospice care is (E)(K4)

A. to preserve life and recognize near death as a normal process

B. not to accelerate or delay death

C. control pain and psychosomatic problems

D. provide a support system to help families deal with bereavement and psychological comfort

E. Respect life, accept death, and assist the child to pass away peacefully and with dignity

5. The principles of hospice care do not include (B)(K5)

A. caring for people, respect for people, and human-centeredness as a guideline for observing problems and dealing with them

B. delaying death

C. Respecting the legitimate wishes of the terminally ill child, providing comprehensive physical, psychological, social and spiritual care for the child and grief counseling for the family

D. The main goal is to improve the quality of life of the child at the end of life

E. Provide 24-hour service for children and their families

6. The wrong connotation of hospice care is (D)(K6)

A. Hospice care needs to provide physical, psychological, social and spiritual multi-dimensional whole-person care

B. In addition to caring for the child, hospice must also care for the family and address physical, psychological, and grief issues

C. Hospice staff are required to manage the child throughout the entire process, and also include grief counseling for the family

D. All related to the care of children do not need to join the team services, only rely on a particular specialty can do a good job of hospice care

E. Hospice care is not only the responsibility of medical institutions and nursing homes, but also the responsibility of society as a whole

7. The one that does not belong to WHO's three-step pain relief principle is (C)(K7)

A. administering medication on demand

B. oral administration

C. administered on time

D. stepwise administration

E. Individualized administration

8. belong to the third order of painkillers are (E)(K8)

A.Ibuprofen

B.Dulcolax

C.codeine

D.Tramadol

E. Morphine

9. The incorrect treatment of pain in hospice care is (A)(K9)

A. encourage the child to persist and fight the pain

B. To scientifically and comprehensively assess the intensity and nature of pain

C. The pain nurse should communicate the results of the pain assessment to the physician in a timely manner

D. Pain treatment should be mainly pharmacological, supplemented by non-pharmacological treatment, and follow the "three-step pain treatment principle".

E. Oral medication is recommended.

10. The wrong statement in hospice care for the comfort of the child is (D)(K10)

A. Keep the mouth moist, brush and rinse gently, and eat popsicles or pineapple.

B. Children with mouth ulcers can use an alcohol-free antibacterial mouthwash for routine oral care.

C. Soft, non-rough sponges, washcloths and towels can be used to gently pat dry the skin.

D. The child can be bathed with baby shower gel or soap.

E. For children with protruding bones who are bedridden for a long time, use anti-crush dressings for external application, such as sacrococcygeal.

11. The symptoms that may occur during hospice care for children are (ABCDE) [multiple choice](K11)

A. cough and sputum

B. nausea and vomiting

C. fatigue and weakness

D. Pain

E. difficulty breathing

12. The practice of hospice care mainly includes (ABCD) [multiple choice](K12)

A.symptom management

B. comfort care

C. Psychological support

D. Humanistic care

E. None of the above

13. The characteristics of hospice care include (ABCDE) [multiple choice](K13)

A. Hospice care is mainly to control pain and discomfort and improve the quality of life of the child

B. Hospice care emphasizes the care of the child and family

C. Services are provided by a team of disciplines working together to provide whole-person, whole-family, whole-team, whole-community care

D. Hospice services are active and can be used early in the disease process

E. Hospice services include symptom control, comfort care, psychological support and humanistic care

14. The goal of hospice care is to (ABCDE) [multiple choice](K14)

A. reduce the suffering of the child

B. maintain the child's dignity

C. help the child pass away peacefully

D. Reduce the burden of bereaved parents

E. All of the above

15. What hospice care has taught us is that (AD) [multiple choice](K15)

A. Hospice care has helped us understand what medicine can and cannot do, and has taught me that medicine has limitations and is not omnipotent

B. Hospice makes us fear death

C. Hospice has taught us that the pursuit of length of life is meaningless

D. Hospice care has helped us to develop a correct attitude toward the end of life: face it openly and end it quietly

E. It is pointless for the terminally ill to suffer, so they should use methods that can relieve their pain immediately

**Hospice-related beliefs/attitudes**

[A total of 18 questions, all single choice]

1. Do you think hospice care is a meaningful thing to do? ()(T1)

A.Strongly agree (5 points)

B.Agree more

C.Not sure

D.Basically disagree

E.Very disagree (1 point)

2. Do you think death is something that runs naturally? ()(T2)

A.Strongly agree

B.Somewhat agree

C.Not sure

D.Basically disagree

E.Strongly disagree

3. Do you think your hospital has sufficient conditions to carry out hospice care at present? ()(T3)

A.Strongly agree

B.Somewhat agree

C.Not sure

D.Basically disagree

E.Strongly disagree

4. Do you think health care workers should avoid talking about death with terminally ill children? ()(T4)

A.Strongly agree

B.Somewhat agree

C.Not sure

D.Basically disagree

E.Very much disagree

5. When the child asks: Am I going to die? Do you suggest changing the topic to something happy? ()(T5)

A.Strongly agree

B. Agree more

C.Not sure

D.Basically disagree

E.Strongly disagree

6. Do you think the family should be actively involved in the child's life care? ()(T6)

A.Strongly agree

B.Agree more

C.Not sure

D.Basically disagree

E.Strongly disagree

7. Do you think it is important to care for the child's family both during and after the child's death? ()(T7)

A.Strongly agree

B.Agree somewhat

C. Not sure

D.Basically disagree

E.Strongly disagree

8. Do you think family members should care for and help the terminally ill child so that he or she can better spend the remaining time of life? ()(T8)

A.Strongly agree

B.Agree more

C.Not sure

D.Basically disagree

E.Strongly disagree

9. Do you think the family's attitude and behavior toward the terminally ill child should remain as normal as possible? ()(T9)

A.Strongly agree

B.Somewhat agree

C.Not sure

D.Basically disagree

E.Strongly disagree

10. Do you think the leading decision maker at the end of life is the child and the family? ()(T10)

A.Strongly agree

B.Somewhat agree

C.Not sure

D.Basically disagree

E.Strongly disagree

11. Do you think it is in the dying child's own interest to use words to express his or her feelings? ()(T11)

A.Strongly agree

B.Agree more

C.Not sure

D.Basically disagree

E.Strongly disagree

12. Do you think we should inform the terminally ill child of his or her condition truthfully? ()(T12)

A.Strongly agree

B.Somewhat agree

C.Not sure

D.Basically disagree

E.Strongly disagree

13. Do you think health care workers should take courses related to death education or hospice care? ()(T13)

A.Strongly agree

B.Somewhat agree

C.Not sure

D.Basically disagree

E.Strongly disagree

14. The cost of hospice care at the child's home should be included in the medical insurance reimbursement ()(T14)

A.Strongly agree

B.Somewhat agree

C.Not sure

D.Basically disagree

E.Strongly disagree

15. The government currently attaches great importance to hospice care for children ()(T15)

A.Strongly agree

B.Somewhat agree

C.Not sure

D.Basically disagree

E. strongly disagree

**Hospice-related behaviors**

[A total of 20 questions, all single choice]

1. In your work, can you choose effective assessment tools to dynamically and comprehensively assess the pain level of terminally ill children, provide pain education to terminally ill children or their families, instruct on the correct medication, and explain the precautions and adverse effects of taking medication? ()(B1)

A.Rarely (1 point)

B.Occasionally

C.Sometimes

D.Often

E.Always（5 points)

2. Do you introduce knowledge about oral care to terminally ill children or their families at work, and provide targeted guidance according to the problems of terminally ill children?(B2)

A.Rarely

B.Occasionally

C.Sometimes

D.Often

E.Always

3. Can you guide and assist terminally ill children and their families in their work to frequently check their own skin, keep their skin and bed units clean and hygienic, promote comfort, and prevent pressure sores and other complications? ()(B3)

A.Rarely

B.Occasionally

C. Sometimes

D. Often

E.Always

4. Can you check the nutritional status of terminally ill children in a timely and correct manner, select appropriate dietary treatment and care plans, and improve the nutritional status of terminally ill children in your work? ()(B4)

A.Rarely

B.Occasionally

C. Sometimes

D. Often

E. Always

5. You are able to accurately assess the respiratory status of the terminally ill child, select the appropriate position according to the condition, improve cough and sputum, and instruct nebulized inhalation ()(B5)

A.Rarely

B.Occasionally

C. Sometimes

D.Often

E. Always

6. Can you take the initiative in your work to understand the sleeping condition of the terminally ill child and take measures (such as controlling pain, ensuring smooth breathing, relaxing joints and muscles, etc.) to promote the sleep of the terminally ill child? ()(B6)

A. Rarely

B.Occasionally

C. Sometimes

D. Often

E.Always

7. Do you actively listen to your terminally ill child and let him/her fully express his/her inner feelings?(B7）

A.Rarely

B.Occasionally

C.Sometimes

D.Often

E.Always

8. Can you use patient, encouraging, and instructive words with the terminally ill child and use therapeutic touch when appropriate?

therapeutic touch? (B8)

A. Rarely

B. Occasionally

C. Sometimes

D.Often

E.Always

9. Do you take the initiative to think and handle things from the perspective of the dying child or family? ()(B9)

A.Very seldom

B.Occasionally

C.Sometimes

D.Often

E. Always

10. Do you actively assess the attitude of the terminally ill child or family toward death? ()(B10)

A.Rarely

B.Occasionally

C.Sometimes

D.Often

E.Always

11. Do you help terminally ill children or their families gain knowledge about death and dying and understand death correctly? ()(B11)

A. Rarely

B. Occasionally

C. Sometimes

D. Often

E.Always

12. Do you help terminally ill children/families to find the causes of anger and anxiety, collect their psychosocial information, and adopt appropriate methods to psychologically guide them? ()(B12)

A.Rarely

B.Occasionally

C. Sometimes

D. Often

E.Always

13. Would you encourage the terminally ill child/family to set realistic and achievable goals and assist them in accomplishing their wishes? ()(B13)

A.Rarely

B.Occasionally

C.Sometimes

D. Often

E. Always

14. Can you encourage family members to spend more time with the terminally ill child and promote effective communication between them and the terminally ill child?(B14)

A. Rarely

B. Occasionally

C. Sometimes

D.Often

E. Always

15. Can you meet the special needs of the terminally ill child/family as much as possible according to the race, culture, beliefs and values of the terminally ill child/family? ()(B15)

A.Rarely

B. Occasionally

C. Sometimes

D. Often

E.Always

16. Do you respect the rights of the terminally ill child and ask for the views of the terminally ill child/family when developing a care plan? ()(B16)

A.Very seldom

B.Occasionally

C. Sometimes

D. Often

E.Always

17. Can you assess the psychological state of the dying child's family, accompany them, comfort them, listen to them, and encourage them to express their grief? ()(B17)

A. Rarely

B. Occasionally

C. Sometimes

D. Often

E. Always

18. Can you encourage and assist families to find appropriate ways to say goodbye to their terminally ill children before they die? ()(B18)

A.Rarely

B. Occasionally

C. Sometimes

D. Often

E. Always

19. Can you encourage family members to send their condolences through appropriate means (such as writing letters to their deceased loved ones)? ()(B19)

A.Very seldom

B. Occasionally

C. Sometimes

D. Often

E.Always

20. After the death of the child, did you use telephone, letters, and the Internet to follow up and support the bereaved person to express your sympathy and care so that he or she can resume his or her social role as soon as possible? ()(B20)

A.Rarely

B.Occasionally

C. Sometimes

D. Often

E.Always
